# Association of Substance Use With Behavioral Adherence to Centers for Disease Control and Prevention Guidelines for COVID-19 Mitigation: Cross-sectional Web-Based Survey

**DOI:** 10.2196/29319

**Published:** 2021-11-09

**Authors:** Mollie A Monnig, Hayley Treloar Padovano, Alexander W Sokolovsky, Grace DeCost, Elizabeth R Aston, Carolina L Haass-Koffler, Claire Szapary, Patience Moyo, Jaqueline C Avila, Jennifer W Tidey, Peter M Monti, Jasjit S Ahluwalia

**Affiliations:** 1 Department of Behavioral and Social Sciences Brown University Providence, RI United States; 2 Center for Addiction and Disease Risk Exacerbation Brown University Providence, RI United States; 3 Department of Psychiatry and Human Behavior Brown University Providence, RI United States; 4 Center for Gerontology and Healthcare Research Department of Health Services, Policy, and Practice Brown University Providence, RI United States

**Keywords:** SARS-CoV-2, novel coronavirus, COVID-19, alcohol use, alcohol drinking, opioid use, stimulant use, nicotine, smoking, survey, substance abuse, addiction, mental health, pandemic

## Abstract

**Background:**

Substance use is a risk factor for COVID-19 infection and adverse outcomes. However, reasons for elevated risk for COVID-19 in substance users are not well understood.

**Objective:**

The aim of this study was to evaluate whether alcohol or other drug use is associated with adherence to Centers for Disease Control and Prevention (CDC) guidelines for COVID-19 mitigation. Preregistered analyses tested the hypothesis that greater use of alcohol and other drugs would be associated with lower CDC guideline adherence. A secondary objective was to determine whether substance use was associated with the likelihood of COVID-19 testing or outcome.

**Methods:**

A cross-sectional web-based survey was administered to a convenience sample recruited through Amazon’s Mechanical Turk platform from June 18 to July 19, 2020. Individuals aged 18 years or older and residing in Connecticut, Massachusetts, New Jersey, New York, or Rhode Island were eligible to participate. The exposure of interest was past 7-day use of alcohol, cigarettes, electronic cigarettes, cannabis, stimulants, and nonmedical opioids. The primary outcome was CDC guideline adherence measured using a scale developed from behaviors advised to reduce the spread of COVID-19. Secondary outcomes were likelihood of COVID-19 testing and a positive COVID-19 test result. All analyses accounted for the sociodemographic characteristics.

**Results:**

The sample consisted of 1084 individuals (mean age 40.9 [SD 13.4] years): 529 (48.8%) men, 543 (50.1%) women, 12 (1.1%) other gender identity, 742 (68.5%) White individuals, 267 (24.6%) Black individuals, and 276 (25.5%) Hispanic individuals. Daily opioid users reported lower CDC guideline adherence than nondaily users (B=–0.24, 95% CI –0.44 to –0.05) and nonusers (B=–0.57, 95% CI –0.76 to –0.38). Daily alcohol drinkers reported lower adherence than nondaily drinkers (B=–0.16, 95% CI –0.30 to –0.02). Nondaily alcohol drinkers reported higher adherence than nondrinkers (B=0.10, 95% CI 0.02-0.17). Daily opioid use was related to greater odds of COVID-19 testing, and daily stimulant use was related to greater odds of a positive COVID-19 test.

**Conclusions:**

In a regionally-specific, racially, and ethnically diverse convenience sample, adults who engaged in daily alcohol or opioid use reported lower CDC guideline adherence for COVID-19 mitigation. Any opioid use was associated with greater odds of COVID-19 testing, and daily stimulant use was associated with greater odds of COVID-19 infection. Cigarettes, electronic cigarettes, cannabis, or stimulant use were not statistically associated with CDC guideline adherence, after accounting for sociodemographic covariates and other substance use variables. Findings support further investigation into whether COVID-19 testing and vaccination should be expanded among individuals with substance-related risk factors.

## Introduction

The use of alcohol, tobacco, and other drugs has been identified as a risk factor for infection with severe acute respiratory syndrome coronavirus 2 (SARS-CoV-2), the virus responsible for the COVID-19 pandemic [1,2]. Substance use may increase risk through both biological and behavioral pathways [3]. First, chronic use compromises the immune system and major organs, including cardiac, pulmonary, and renal systems [4,5]. Second, potential behavioral pathways for COVID-19 infection include decreased inhibition, increased risk-taking behavior, and competing contingencies (eg, perceived need to obtain and use substances despite risk of exposure) [6]. A recent study found that substance use disorder diagnosis was associated with greater risk of coronavirus infection (adjusted odds ratio 8.7) and higher death rates [1]. While this finding may be partially explained by higher rates of medical comorbidities that confer increased risk for and severity of COVID-19 among individuals with substance use disorder, behavioral factors were not assessed [1].

Emerging data indicate changes in substance use and related problems in the United States during the pandemic. In a Centers for Disease Control and Prevention (CDC) survey, 13% of US adults reported initiating or increasing substance use to deal with COVID-19–related stress [7]. Another representative survey of US adults reported a 27% increase in average drinks per day and a 26% increase in frequency of binge drinking [8]. Smoking in young adults has shown signs of increased quit rates or reduced frequency overall, but increased quantity on use days [9,10]. Perhaps the starkest indicator is the increase in overdose deaths involving opioids, cocaine, and methamphetamine in 2020 [11,12].

The CDC recommends several behaviors to mitigate the spread of SARS-CoV-2, including avoiding close contact with others outside the household, handwashing, and wearing a face covering [13]. States have employed diverse mandates to control the spread of COVID-19, including limitations on social gatherings, business closures, and stay-at-home orders. Adherence to CDC guidelines and state mandates may be affected by substance use. For example, young adults under stay-at-home orders reported a higher number of in-person contacts outside the household on days they consumed alcohol [14]. Although other studies have assessed factors associated with adherence to COVID-19 mitigation behaviors, most have not addressed substance use [15,16]. Because the pandemic itself appears to be associated with increases in some types of substance use, understanding whether substance use is associated with lower adherence to CDC mitigation guidelines is pressing. Finding an association between substance use and suboptimal preventive behaviors would help to identify specific at-risk groups and to generate hypotheses on mechanisms underlying nonadherence to guidelines.

The objective of this study was to investigate links between use of specific substances and behavioral adherence to CDC guidelines in a sample of US adults surveyed in June and July 2020. Our main hypothesis predicted that greater substance use would be associated with lower CDC guideline adherence after accounting for demographic and contextual factors that have been associated with CDC guideline adherence or related health behaviors in previous studies, such as age and income [15-19]. Given that lower guideline adherence would have an expected association with COVID-19 exposure and infection, secondary analyses tested whether substance use was associated with the likelihood of receiving a COVID-19 test or with the outcome of testing.

## Methods

### Study Design and Data Source

This study was a deidentified, web-based, cross-sectional survey with a nonprobability sample. Participants were recruited using Amazon’s Mechanical Turk (MTurk) platform. MTurk is a crowdsourcing platform with a built-in survey administration system that allows researchers access to a pool of >200,000 potential respondents, producing data of quality equal to or better than professionally sourced panels [20-23]. Our study focused on 5 northeastern states (Connecticut, Massachusetts, New Jersey, New York, and Rhode Island) that had the highest numbers of COVID-19 cases and deaths per capita in the United States at the time [24]. A pilot was released on May 27, 2020, for survey refinement. The final survey was released from June 18 to July 19, 2020, at which time the 5 states had restrictions on business capacity and social gatherings (see Table S1 in Multimedia Appendix 1). This study was an initiative of the Center for Addiction and Disease Risk Exacerbation to examine the link between substance use and disease [25].

### Participants

Eligibility criteria were as follows: ≥18 years of age, residing in an eligible state (Connecticut, Massachusetts, New Jersey, New York, or Rhode Island), and holding an active MTurk account. Quotas based on age, gender, race, and ethnicity were used to ensure a diverse sample. Black and Hispanic individuals were oversampled owing to overwhelming evidence that such individuals have been disproportionately affected by the pandemic [26-30]. Race and ethnicity quotas were as follows: 40% non-Hispanic White, 25% Hispanic non-Black, 25% Black any ethnicity, and 10% non-Hispanic non-White. Within each racial/ethnic group, the following age quotas were applied: 10%, 18-25 years; 20%, 25-35 years; 20%, 35-45 years; 25%, 45-55 years; and 25%, ≥55 years. Percentages were allocated to each age range (which are preset in the MTurk system) to ensure broad representation of ages and specifically to avoid the underrepresentation of older adults often observed in web-based survey studies [31]. Within each cell type based on race/ethnicity and age, quotas stipulated equal numbers of men and women. Interested individuals completed a brief screening survey to assess demographics and were directed to the main survey if their quotas were not already filled. Participants provided written informed consent before beginning the survey. Participants were paid US $10 upon completion. This study was reviewed by the Brown University Institutional Review Board and was determined to be exempt from requiring Institutional Review Board approval as a minimal risk study per federal regulations.

### Measurements

#### Sociodemographic Characteristics

Demographic characteristics included age, sex, gender identity, highest level of education, annual household income, and home ownership. These variables have demonstrated associations with adherence to COVID-19 prevention/mitigation behaviors in US adults in previous research [15-18] aside from home ownership, a proxy for wealth, which was chosen due to relations with health behaviors in other contexts [19]. Race and ethnicity were assessed with a two-item measure from the 2020 Household Pulse Survey [32]. Individuals indicated whether they were Hispanic, Latino, or of Spanish origin, and selected all races that applied. Participants were asked essential worker status, defined as “someone whose work is critical to business operations and/or meeting basic human needs and is required to attend work during the COVID pandemic” (yes/no/not sure).

#### Primary Outcome: CDC Guideline Adherence

The primary outcome was measured using a self-report questionnaire that we developed from the CDC’s recommendations for behaviors in which the public should engage to mitigate COVID-19 transmission (see Table 1). Participants rated how often they engaged in 13 recommended behaviors during the past 4 weeks on a scale from 0 (rarely or never) to 3 (always), with higher scores representing higher adherence. Parallel analysis and inspection of factor loadings supported use of the total item average as a unidimensional construct reflecting CDC guideline adherence. The internal consistency reliability was excellent (Cronbach α .91).

**Table 1:** Primary outcome: Centers for Disease Control and Prevention guideline adherence measure.^a^

During the past 4 weeks, how often did you…	Always	Usually	Sometimes	Rarely or Never
Wash your hands often with soap and water for at least 20 seconds especially after you have been in a public place, or after blowing your nose, coughing, or sneezing?				
Use hand sanitizer that contains at least 60% alcohol when soap and water was not readily available?				
Avoid touching your eyes, nose, and mouth?				
Avoid close contact with people who are sick?				
Remain at least 6 feet away from other people when in public?				
Stay home as much as possible?				
Use a cloth face cover over your nose and mouth when in public?				
Cover your mouth and nose with a tissue or use the inside of your elbow when you coughed or sneezed?				
Throw used tissues in the trash?				
Immediately wash your hands with soap and water for at least 20 seconds after coughing or sneezing?				
Clean and disinfectant frequently touched surfaces in your home (eg, tables, doorknobs, light switches, countertops, desks, phones, toilets, faucets)?				
Use detergent or soap and water to clean dirty surfaces before disinfection?				
When cleaning surfaces, how often did you use any of the following: a diluted household bleach, a solution that was at least 70% alcohol, or another EPA^b^-registered household disinfectant?				

^a^Participants rated how often they engaged in the recommended behaviors in this table during the past 4 weeks on a scale from 0 (rarely or never) to 3 (always), with higher scores representing higher adherence.

^b^EPA: Environmental Protection Agency.

#### Secondary Outcome: COVID-19 Exposure and Testing

COVID-19 testing and test results were the secondary outcomes. Testing history was assessed by asking “Have you been tested for the novel coronavirus or COVID-19?” (yes/no/not sure). Answering “yes” prompted these follow-up questions: “Have you had a nose swab test for the virus that causes COVID-19?” and “Have you had a blood test to see if you already had the virus (“serology”)?” Each of these were followed with the question, “If yes, what was your result?”

#### Substance Use

Substance use was recorded using the Timeline Followback method [33] for the 7 days preceding survey completion. If the participant endorsed any use of cigarettes, electronic cigarettes (e-cigarettes), cannabis, alcohol, opioids, or stimulants, the participant was asked to report whether the substance was used on each day in the past week. For opioids only, participants were instructed, “Do not report any drug that was taken as directed by a physician.” Past 7-day frequencies of alcohol, cigarette, e-cigarette, cannabis, stimulant, and nonmedical opioid use were recoded to reflect no use, 1-6 days of use, and daily use.

### Statistical Analysis

The analytic plan was preregistered and can be accessed online [34]. A descriptive analysis assessed the univariate distributions of demographic characteristics, substance use variables, COVID-19 testing, and the CDC guideline adherence outcome. Continuous data were reported as mean (SD), and categorical variables were reported as count (percentage). Distributional properties of outcomes informed model selection. Code and data analysis were generated using SAS software, Version 9.4 for Windows (Copyright 2016 by SAS Institute Inc), and R version 4.0.3 [35]. Independent variables accounting for CDC guideline adherence score were evaluated using a general linear model with a continuous outcome and categorical and continuous independent variables. Preregistered covariates tested for inclusion included age (continuous), education (high school or less [reference], some college/1-year degree, college graduate/4-year degree, graduate or professional degree), gender (cisgender female [reference], cisgender male, other gender identity, or prefer not to answer), race (White [reference], Black or African American, Asian, other or more than one racial identity), ethnicity (not Hispanic or Latino [reference], Hispanic or Latino), essential worker status (not essential worker or unsure [reference], essential worker), income (8 ordered categories, treated as continuous), household size (continuous, truncated at 6 maximum), dwelling ownership (no ownership [reference], 1=ownership), and COVID-19 test (no test or unsure [reference]; yes, test negative; yes, test positive). Covariates that were not related to CDC guideline adherence in omnibus Type III sums of squares tests were removed and models retested. Next, focal substance use variables reflecting no use, 1-6 days of use, and daily use of alcohol, cigarettes, e-cigarettes, cannabis, stimulants, and nonmedical opioids were added. Substance use variables that were not related to CDC guideline adherence were removed and models retested. Follow-up two-sided *t* tests compared daily use to 1-6 day and no use categories and 1-6 days of use to no use. Adjusted *R^2^* and *ΔR^2^* evaluated the proportion of variability in CDC guideline adherence accounted for by variables included in the models.

A secondary set of logistic regression models tested associations of covariates and substance use variables with the likelihood of COVID-19 testing and results. An initial logistic model evaluated the likelihood of COVID-19 testing (0=no test or unsure, 1=test). Differences in testing likelihood according to substance use frequency prompted post hoc analyses on possible explanatory factors. The post hoc analyses used independent two-sided *t* tests for continuous variables and Pearson *χ*^2^ tests for categorical variables. A subset analysis among those who reported a COVID-19 test evaluated the likelihood of a positive test result (0=negative, 1=positive). Covariates that were not related to COVID-19 testing or COVID-19 test results in omnibus Type III sums of squares tests were removed and models retested.

## Results

### Demographic Characteristics of the Participants

**Figure 1:**
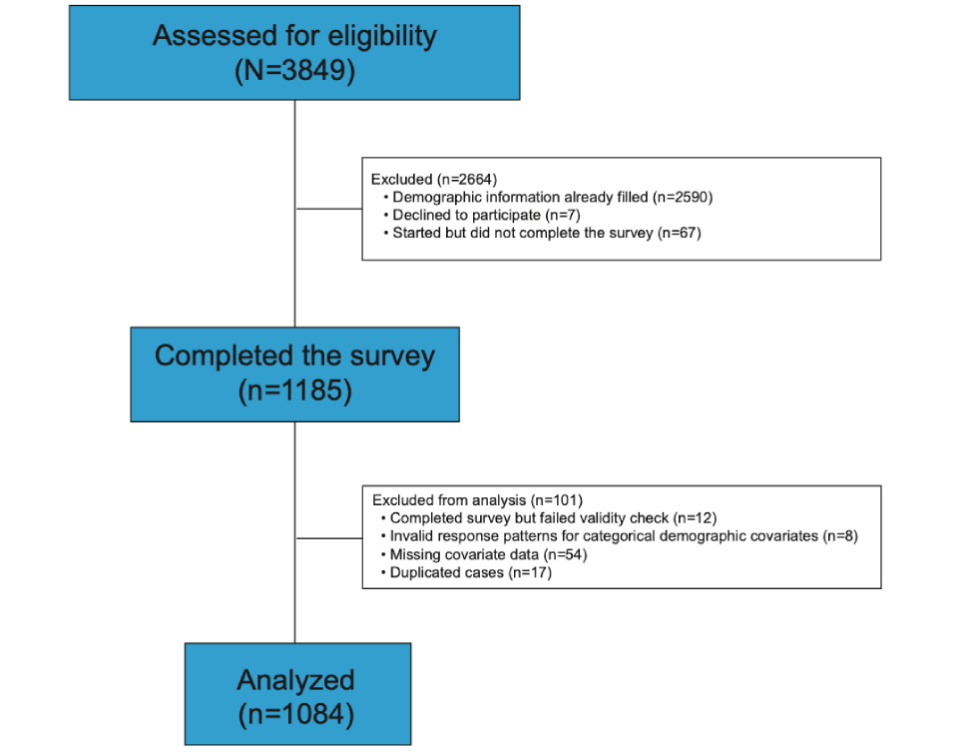
Sample selection of residents of Connecticut, Massachusetts, New Jersey, New York, and Rhode Island for a web-based survey of health behaviors and Centers for Disease Control and Prevention guideline adherence in June-July 2020.

Of the 3849 individuals who were assessed for eligibility, 1185 completed the entire survey (see Figure 1). Data sets were excluded from the analysis if the participant did not pass at least 2 of the 3 validity checks embedded in the survey, for example, Please select the response “strongly agree” (n=12) or if the participant endorsed implausible or mutually exclusive responses on demographic items (n=18). Duplicate data (ie, multiple responses from the same individual) were identified in 17 cases and were removed from the data set. Final models had 1084 participants after excluding participants with missing data for covariates. Demographic information for the evaluable data set including 1084 participants is provided in Table 2. Half of the study sample was males, and the mean age was 40.9 years. The majority of the sample consisted of Whites and had a college degree. Black individuals made up 24.6% (267/1084) of the sample and 25.5% (276/1084) of the participants identified as Hispanic. Approximately one-quarter of the sample had a history of COVID-19 testing. Of those who received a test, 15.8% (44/279) had a positive result. Of the 1084 participants, 700 (64.6%) reported substance use in the past 7 days. Cigarettes were the most common substance used daily, followed by alcohol and opioids. One-third of the sample reported nondaily alcohol use. The majority reported monosubstance use (382/1084, 35.2%), and alcohol was the most common single substance used (252/1084, 23.2%). Polysubstance use (2 or more substances) was reported by 318 individuals (29.3%). The most common combinations of substances were alcohol and opioids (41/1084, 3.8%), alcohol and cigarettes (36/1084, 3.3%), opioids and cigarettes (35/1084, 3.2%), and alcohol and marijuana (23/1084, 2.1%). Other polysubstance combinations were reported by <2% of the sample (<22 individuals per cell).

**Table 2:** Characteristics of the participants (N=1084) in the web-based survey of health behaviors and Centers for Disease Control and Prevention guideline adherence conducted in June-July 2020.

Variable				Value		Recode information	
Age (years), mean (SD)				40.9 (13.4)		None	
**Sex (assigned at birth), n (%)**							We created a combined sex and gender variable recoded to cisgender male if sex=“male” and gender=“man” and no other gender endorsed, cisgender female if sex=“female” and gender=“woman” and no other gender endorsed, and other for all other combinations.
	Male		540 (49.8)				
	Female		536 (49.5)				
	Prefer not to answer		8 (0.7)				
**Gender (select all that apply), n (%)**							We created a combined sex and gender variable recoded to cisgender male if sex=“male” and gender=“man” and no other gender endorsed, cisgender female if sex=“female” and gender=“woman” and no other gender endorsed, and other for all other combinations.
	Man		529 (48.8)				
	Woman		543 (50.1)				
	Nonbinary		6 (0.6)				
	Transgender		4 (0.4)				
	Other or prefer not to answer		9 (0.8)				
**Race (select all that apply), n (%)**							We created 4 race categories recoded to White if race=“White” and no other race endorsed, Black or African American if race=“Black or African American” and no other race endorsed, Asian if race=any of “Asian Indian | Chinese | Filipino | Japanese | Korean | Vietnamese | Other Asian” and no other race endorsed; and Other Race or More Than One for all other endorsed categories.
	White		742 (68.5)				
	Black or African American		267 (24.6)				
	American Indian or Alaskan Native		26 (2.4)				
	Asian Indian		34 (3.1)				
	Chinese		31 (2.9)				
	Filipino		6 (0.5)				
	Japanese		3 (0.3)				
	Korean		7 (0.6)				
	Vietnamese		1 (0.1)				
	Other Asian		15 (1.4)				
	Native Hawaiian		2 (0.2)				
	Guamanian or Chamorro		1 (0.1)				
	Other Pacific Islander		5 (0.5)				
**Ethnicity (select all that apply), n (%)**							We dichotomized ethnicity to Not of Hispanic, Latino/Latina, or Spanish origin, and all other endorsed categories
	Not of Hispanic, Latino/Latina, or Spanish Origin		808 (74.5)				
	Mexican, Mexican American, or Chicano/Chicana		76 (7.0)				
	Puerto Rican		42 (3.9)				
	Cuban		14 (1.3)				
	Another Hispanic, Latino/Latina, or Spanish origin		155 (14.3)				
**Education, n (%)**							None
	College graduate		547 (50.5)				
	Some college		215 (19.8)				
	Professional degree		215 (19.8)				
	High school or lower		107 (9.9)				
**Annual household income, n (%)**							Eight ordered categories were treated continuously.
	Less than US $25,000		123 (11.4)				
	US $25,000-$34,999		122 (11.3)				
	US $35,000-$49,999		152 (14.0)				
	US $50,000-$74,999		287 (26.5)				
	US $75,000-$99,999		188 (17.3)				
	US $100,000-$149,999		139 (12.8)				
	US $150,000-$199,999		39 (3.6)				
	US $200,000 and above		34 (3.1)				
Household size				3.06 (1.33)		None	
Dwelling ownership (yes)				507 (46.8)		None	
**Essential worker, n (%)**							Dichotomized to Yes and No or unsure.
	No		641 (59.1)				
	Yes		388 (35.8)				
	Unsure		55 (5.1)				
Centers for Disease Control and Prevention adherence				2.20 (0.62)		None	
**COVID-19 testing history, n (%)**							None
	No test or unsure		805 (74.3)				
	Yes		279 (25.7)				
**COVID-19 test result (n=279), n (%)**							Either a positive nasal or blood test was recoded as a positive COVID-19 test result.
	Negative		235 (84.2)				
	Positive		44 (15.8)				
**Substance use, n (%)**							
	**Cigarette use (past 7 days)**					Recoded from Timeline Followback	
		None	865 (79.8)				
		Nondaily (1-6 days)	66 (6.1)				
		Daily (7 days)	153 (14.1)				
	**Electronic cigarette use (past 7 days), n (%)**					Recoded from Timeline Followback	
		None	995 (91.8)				
		Nondaily (1-6 days)	39 (3.6)				
		Daily (7 days)	50 (4.6)				
	**Cannabis (past 7 days), n (%)**					Recoded from Timeline Followback. Includes use of any form of cannabis	
		None	973 (89.8)				
		Nondaily (1-6 days)	49 (4.5)				
		Daily (7 days)	62 (5.7)				
	**Alcohol (past 7 days), n (%)**					Recoded from Timeline Followback	
		None	643 (59.3)				
		Nondaily (1-6 days)	356 (32.8)				
		Daily (7 days)	85 (7.8)				
	**Opioids (past 7 days), n (%)**					Recoded from Timeline Followback. Includes use of any opioids	
		None	858 (79.2)				
		Nondaily (1-6 days)	180 (16.6)				
		Daily (7 days)	46 (4.2)				
	**Stimulants (past 7 days), n (%)**					Recoded from Timeline Followback	
		None	1015 (93.6)				
		Nondaily (1-6 days)	42 (3.9)				
		Daily (7 days)	27 (2.5)				

### CDC Guideline Adherence

Covariates tested for inclusion are reported in Table 2. The following variables were statistically related to CDC guideline adherence and retained in the model: gender, ethnicity, essential worker status, income, and COVID-19 test. Covariates accounted for 7.1% of the variance in CDC guideline adherence (*R*^2^=0.071, adjusted *R*^2^=0.057). Unadjusted relations of substance use variables with CDC guideline adherence scores are provided in Table S2 in Multimedia Appendix 1. Cigarette, alcohol, opioid, and stimulant use were related to CDC guideline adherence scores in bivariate models. In the multivariable model including all substance use variables and significant covariates from Table 1, only alcohol consumption and opioid use were statistically related to CDC guideline adherence. The results of the final model are reported in Table 3. Participants who reported daily opioid use reported lower CDC guideline adherence than participants who reported 1-6 opioid use days or no opioid use. Likewise, participants who reported 1-6 opioid use days reported lower adherence than those who reported no use. Participants who reported 1-6 drinking days reported higher CDC guideline adherence than participants who reported daily drinking or no drinking, whereas daily drinkers and nondrinkers did not differ statistically on CDC adherence. The final model retaining only significant covariates, alcohol use, and opioid use accounted for 12.9% of the variance in CDC guideline adherence (*R*^2^=0.129, adjusted *R*^2^=0.120), where the covariate model reported above had accounted for 7.1% of the variance in CDC guideline adherence. Thus, when considering influences on CDC adherence, alcohol and opioid use contributed nearly as much to understanding adherence as did the combination of gender, ethnicity, essential worker status, income, and COVID-19 test.

**Table 3:** General linear model of associations between substance use and Centers for Disease Control and Prevention guideline adherence, accounting for covariates in the full sample of survey participants (N=1084).

Variable		Unstandardized estimate	95% CI
Intercept		2.26	2.19 to 2.37
**Gender**			
	Cisgender female	Ref^a^	Ref
	Cisgender male	−0.17	−0.24 to −0.10
	Other gender identity or prefer not to answer	−0.42	−0.62 to −0.21
Hispanic or Latino (Ref: No)		−0.05	−0.13 to 0.03
Essential worker (Ref: No)		−0.04	−0.13 to 0.04
Income		0.02	0.004 to 0.04
**COVID-19 test**			
	No test or unsure	Ref	Ref
	Negative test	0.10	0.01 to 0.19
	Positive test	−0.02	−0.21 to 0.17
**Alcohol use (past 7 days)**			
	Daily versus none	−0.07	−0.20 to 0.06
	1-6 days versus none	0.10	0.02 to 0.17
	Daily versus 1-6 days^b^	−0.16	−0.30 to −0.02
**Opioid use**			
	Daily versus none	−0.57	−0.76 to −0.38
	1-6 days versus none	−0.32	−0.43 to −0.22
	Daily versus 1-6 days^b^	−0.24	−0.44 to −0.05

^a^Ref: reference category.

^b^Indicates reference groups and additional models for substance use variables where reference groups were switched to allow for additional comparisons.

### COVID-19 Testing

The relation of CDC guideline adherence with COVID-19 testing was also evaluated and found to be not statistically related. Of the covariates in Table 2, the following were related to the likelihood of COVID-19 testing: age, education, race, ethnicity, essential worker status, income, and household size. Of substance use variables, only opioid use was statistically related to testing likelihood. The results of the final model are reported in Table 4. For a daily opioid user, the odds of reporting COVID-19 testing were 3.35 times as large as the odds for a nonuser and 1.61 times as large as the odds for a participant who used opioids on 1-6 days.

**Table 4:** Logistic regression model of associations between substance use and any COVID-19 testing, accounting for covariates in the full sample of survey participants (N=1084).^a^

Variable		Unadjusted prevalence and percentage testing within category, n (%)	Odds ratio (95% CI)
Age		N/A^b^	1.01 (1.00-1.02)
**Education**			
	*Graduate or professional degree*	64 (29.8)	Ref^c^
	College graduate/4-year degree	162 (29.6)	0.70 (0.47-1.05)
	High school or less	19 (17.8)	0.45 (0.23-0.86)
	Some college/2-year degree	34 (15.8)	0.41 (0.24-0.69)
Hispanic or Latino		118 (42.8)	1.67 (1.18-2.37)
*Not Hispanic or Latino*		161 (19.9)	Ref
**Racial identity**			
	White	165 (23.2)	Ref
	Asian	9 (11.7)	0.66 (0.31-1.44)
	Black or African American	89 (37.2)	1.60 (1.12-2.30)
	Other or more than one	16 (27.6)	1.26 (0.63-2.52)
Essential worker		164 (42.3)	2.22 (1.60-3.09)
*Not essential worker*		115 (16.5)	Ref
Income		N/A	0.85 (0.77-0.94)
Household size		N/A	1.33 (1.17-1.50)
**Opioid use**			
	Daily versus none	32^d^ (69.6^d^)	3.35 (1.63-6.86)
	1-6 days versus none	93^e^ (51.7^e^)	2.08 (1.39-3.11)
	*Daily versus 1-6 days*	*154*^f^*(18.0*^f^)	*1.61 (0.78-3.34)*

^a^Italicized text indicates reference groups and additional models for substance use variables where reference groups were switched to allow for additional comparisons. Unadjusted prevalence values are provided for categorical variables only.

^b^N/A: not applicable.

^c^Ref: reference category.

^d^Daily.

^e^1-6 days.

^f^None.

Following the finding that daily and nondaily opioid users were more likely to receive a COVID-19 test, we conducted 2 post hoc analyses on factors that might plausibly lead to higher testing rates in opioid users. First, we asked whether opioid users had significantly higher rates of comorbid medical conditions known to increase the risk of COVID-19 in their households by comparing the count of how many of the following conditions were endorsed by the participants: autoimmune disease, cardiovascular disease, cerebrovascular disease, chronic lung disease, diabetes, immune compromise, or kidney disease. Independent *t* tests showed no difference in this count of medical conditions between daily users versus nonusers (t_902_=–0.016, *P*=.98) or between nondaily users versus nonusers (t_1036_=–1.333, *P*=.18). Second, we asked whether engagement in medication-assisted treatment (MAT) for opioid use disorder was associated with a higher likelihood of receiving a COVID-19 test. In the total sample, MAT was endorsed by 61% (28/46) of daily opioid users, 32.2% (58/180) of nondaily opioid users, and 2.1% (18/858) of nonusers. Among opioid users, 40.7% (57/140) of opioid users not engaged in MAT received a COVID-19 test, whereas 79% (68/86) of opioid users engaged in MAT received a COVID-19 test. This difference was significant (Pearson *χ*^2^_1_=31.8, *P*<.001). In summary, MAT engagement was associated with higher rates of COVID-19 testing in nondaily and daily opioid users. The likelihood of a positive result was evaluated among the subset who received a COVID-19 test. The following Table 2 covariates were statistically related to a positive result: ethnicity, household size, and dwelling ownership. Of substance use variables, only stimulant use statistically related to testing likelihood. The results of the final model are reported in Table 5. For a daily stimulant user, the odds of reporting a positive COVID-19 test among those who were tested was significantly higher for daily users than that for either nonusers or those who reported use on 1-6 days. There was no statistically significant difference in receiving a positive COVID-19 test for nondaily stimulant users versus nonusers.

**Table 5:** Logistic model of associations of stimulant use with positive COVID-19 test result, accounting for covariates, in the subset of participants reporting a COVID-19 test (n=279).^a^

Variable			Unadjusted prevalence and percentage testing within category, n (%)		Odds ratio (95% CI)
Hispanic or Latino			28 (23.7)		2.00 (1.96-4.19)
*Not Hispanic or Latino*			16 (9.9)		Ref^b^
Household size			N/A^c^		1.49 (1.08-2.07)
Dwelling ownership			15 (11.2)		0.45 (0.21-0.95)
*No dwelling ownership*			29 (20.0)		Ref
**Stimulant use**					
	Daily versus none	12^d^ (70.6^d^)		10.49 (3.19-34.44)	
	1-6 days versus none	5^e^ (18.5^e^)		1.44 (0.49-4.25)	
	Daily versus 1-6 days	27^f^ (11.5^f^)		7.29 (1.66-32.05)	

^a^Italicized text indicates reference groups and additional models for substance use variables where reference groups were switched to allow for additional comparisons. Unadjusted prevalence values are provided for categorical variables only.

^b^Ref: reference category.

^c^N/A: not applicable.

^d^Daily.

^e^1-6 days.

^f^None.

## Discussion

### Principal Results

This study tested the hypothesis that heavier substance use would be associated with lower adherence to CDC guidelines to reduce the spread of COVID-19. Our geographic focus was 5 states that had experienced the highest rates of COVID infection and deaths at the time of the survey [24]. Our hypothesis was partially supported in that daily drinkers reported lower adherence than those who drank 1-6 days per week, and daily opioid users reported lower adherence than those who used opioids 1-6 days per week or not at all. However, we did not observe statistical associations of cigarettes, e-cigarettes, cannabis, or stimulant use with CDC guideline adherence, after accounting for sociodemographic covariates and other substance use variables.

Use of some substances was related to the likelihood either of receiving a COVID-19 test or of receiving a positive test. The odds of COVID-19 testing were 3.35 times as large for daily opioid users as for nonusers and 2.08 times as large for nondaily opioid users as for nonusers. Post hoc analyses showed that opioid users did not have significantly higher rates of comorbid medical conditions than nonusers, but they did report high rates of engagement in MAT for opioid use disorder. In turn, MAT was associated with significantly higher rates of COVID-19 testing in opioid users. Although speculative, it is possible that engagement in MAT increased contact with the health care system and thereby increased testing rates in opioid users. In addition, among those who received a COVID-19 test, odds of a positive test were significantly higher in daily stimulant users than those who used stimulants less frequently or not at all. There is a large body of research linking the use of amphetamine, methamphetamine, and other stimulants to HIV transmission, largely through risky sexual behavior [36-38]. Although sexual contact is not central to COVID-19 transmission, it may be that stimulant users had a riskier pattern of in-person contacts (eg, more frequent or less observant of distancing) that increased their risk of COVID-19 infection.

Cigarette use was associated with CDC guideline adherence in unadjusted analyses; yet, this variable became nonsignificant after adjusting for sociodemographic characteristics and other substance use. This pattern suggests that other characteristics or behaviors associated with cigarette use are driving the finding. Null results observed for e-cigarette, cannabis, and stimulant use should be interpreted within the limits of the study design and sample size. The prevalence of nondaily or daily e-cigarette, cannabis, and stimulant use was less than 6%. For these substances, the study may not have had sufficient power to detect differences in CDC adherence scores.

In the final model, gender, income, and COVID-19 test history were the demographic covariates that remained significant after the inclusion of substance use variables. Women reported higher adherence than men and those with other gender identities, and income showed a positive linear association with adherence. These findings are consistent with previous research [15,17]. Participants with a negative COVID-19 test reported higher adherence than those who had not had a test or were unsure. There was no difference in the adherence between those reporting a negative versus positive test result. The reason for this finding is unclear but may involve higher vigilance in those who had received a negative test result. Although we oversampled Black and Hispanic individuals to ensure representation, neither race nor ethnicity was a significant predictor of CDC guideline adherence after accounting for substance use.

Substance use is a known risk factor for COVID-19 infection [1]. A major implication of our findings is that lower behavioral adherence to CDC guidelines may be one mechanism by which substance use increases risk for COVID-19 infection. These findings should not be used to further stigmatize these individuals, who already face considerable stigma and adversity in social, occupational, and health care settings. Rather, results reinforce the need for outreach efforts and interventions that support behavioral adherence, COVID-19 testing, and vaccination for this population. Limited previous research has addressed this topic. We did not replicate the finding that use of e-cigarettes and combustible cigarettes was associated with COVID-19 testing and positive diagnosis [39]. However, that study was limited to individuals aged 13-24 years, which differs from our adult population.

Interestingly, individuals who drank 1-6 days per week reported higher CDC guideline adherence relative to nondrinkers and daily drinkers who did not differ statistically from each other. This finding may be in line with other observational research linking moderate or occasional alcohol consumption to health behaviors such as physical activity [40,41]. This interpretation is speculative as alcohol quantity was not included in the model, and therefore, nondaily drinkers, nevertheless, may have engaged in heavy episodic drinking. Given the present findings regarding alcohol consumption and COVID-19, further work in this space is warranted.

### Limitations

The limitations of this study include the use of a nonrepresentative convenience sample, self-report data, and a novel CDC adherence scale that has not yet been validated. The survey did not explicitly assess the number or timing of COVID-19 tests that participants had received. Absence of data on the timing of testing is a significant limitation particularly with regard to understanding the relation with substance use. Analyses accounted for frequency but not quantity of substance use or specific patterns of polysubstance use, both of which are potentially important factors that warrant attention in future research. Our findings may not generalize to the US population as a whole. For effects with large confidence intervals, findings should be interpreted as informing directionality for future research rather than representing a reliable estimate of their magnitude in the sampled population.

### Conclusions

In a convenience sample of adults living in the northeastern United States, we found that daily use of alcohol or opioids was significantly associated with lower adherence to CDC guidelines for reducing the spread of COVID-19, after accounting for sociodemographic characteristics and other substance use. However, use of cigarettes, e-cigarettes, cannabis, or stimulants was not associated with adherence. We also found that daily use of nonprescribed opioids was associated with higher odds of COVID-19 testing and that daily use of stimulants was associated with higher odds of a positive COVID-19 test result. The strengths of this study include the calendar-based assessment of several common classes of substances and the racial, ethnic, and age diversity of the sample. The findings of this study point to a need for public health efforts to support behavioral adherence and to expand COVID-19 testing and vaccination in individuals with high levels of alcohol, opioid, or stimulant use.

## Appendix

Multimedia Appendix 1 Supplemental tables.

## Abbreviations

CDC: Centers for Disease Control and Prevention
e-cigarette: electronic cigarette
MAT: medication-assisted treatment
MTurk: Mechanical Turk
