# Supplementary material for: Association of Substance Use With Behavioral Adherence to Centers for Disease Control and Prevention Guidelines for COVID-19 Mitigation: Cross-sectional Web-Based Survey
Source: JMIR Public Health Surveill. 2021 Nov 9;7(11):e29319. doi: 10.2196/29319 (PMC8582757; doi:10.2196/29319)
Supplement: Multimedia Appendix 1 [file publichealth_v7i11e29319_app1.docx]

Table S1. COVID-19 guidelines and restrictions during survey administration in June to July 2020

| State | Date of Study Inclusion | Phase of Reopening at Time of Study Inclusion | Phase Reopening Guidelines | Date Stay-at-Home Order Issued | Date Stay-at-Home Order Lifted |
| --- | --- | --- | --- | --- | --- |
| Connecticut | June 25, 2020 | Phase II | Indoor gatherings <25 people  All businesses operating at 50% capacity | March 28, 2020 | May 20, 2020 |
| Massachusetts | June 25, 2020 | Phase II | Social gatherings <10 people  All businesses operating at 50% capacity | March 24, 2020 | May 18, 2020 |
| New Jersey | July 6, 2020 | Phase II | Indoor gatherings <50  Indoor dining at 25% capacity  Office space at 50% capacity | March 21, 2020 | June 9, 2020 |
| New York | July 6, 2020 | Phase III | Social gatherings <25 people  Indoor dining at 50% capacity (excludes New York City)  Office space at 50% capacity | March 22, 2020 | June 8, 2020^a^ |
| Rhode Island | June 18, 2020 | Phase II | Social gatherings <15 people  Indoor dining at 50% capacity  Office space at 33% capacity | March 28, 2020 | May 8, 2020 |

^a^As issued by Gov. Cuomo, this is the earliest date that allowed certain counties in New York to enter into Phase I of reopening.

| Substance | Use Pattern | | | P value |
| --- | --- | --- | --- | --- |
|  | None | Non-daily | Daily |  |
|  | Mean (SD) | Mean (SD) | Mean (SD) |  |
| Cigarettes | 2.22 (0.58) | 2.04 (0.80) | 2.11 (0.68) | .014 |
| E-cigarettes | 2.21 (0.61) | 2.14 (0.66) | 2.01 (0.68) | .08 |
| Cannabis | 2.19 (0.63) | 2.26 (0.44) | 2.26 (0.58) | .53 |
| Alcohol | 2.16 (0.65) | 2.29 (0.52) | 2.04 (0.71) | <.001 |
| Opioids | 2.28 (0.55) | 1.92 (0.73) | 1.63 (0.71) | <.001 |
| Stimulants | 2.22 (0.61) | 1.82 (0.71) | 1.70 (0.60) | <.001 |

Table S2. Unadjusted relations of substance use variables with Centers for Disease Control and Prevention Guideline Adherence scores in residents of Connecticut, Massachusetts, New Jersey, New York, and Rhode Island in a web-based survey
